# The Effect of the Extraction Conditions on the Antioxidant Activity and Bioactive Compounds Content in Ethanolic Extracts of *Scutellaria baicalensis* Root

**DOI:** 10.3390/molecules29174153

**Published:** 2024-09-01

**Authors:** Małgorzata Dzięcioł, Klaudia Wala, Agnieszka Wróblewska, Katarzyna Janda-Milczarek

**Affiliations:** 1 Department of Chemical Organic Technology and Polymeric Materials, Faculty of Chemical Technology and Engineering, West Pomeranian University of Technology in Szczecin, Piastów Ave. 42, 71-065 Szczecin, Poland; klaudiawala94@gmail.com; 2 Department of Catalytic and Sorbent Materials Engineering, Faculty of Chemical Technology and Engineering, West Pomeranian University of Technology in Szczecin, Piastów Ave. 42, 71-065 Szczecin, Poland; agnieszka.wroblewska@zut.edu.pl; 3 Department of Human Nutrition and Metabolomics, Pomeranian Medical University in Szczecin, 24 Broniewskiego Street, 71-460 Szczecin, Poland

**Keywords:** antioxidant activity, bioactive compounds, Baikal skullcap, ethanolic extracts, DPPH scavenging activity, extraction techniques, GC-MS, *Scutellaria baicalensis*, total phenolic content

## Abstract

Ethanolic extracts of Baikal skullcap (*Scutellaria baicalensis*) root were obtained using various techniques, such as maceration, maceration with shaking, ultrasound-assisted extraction, reflux extraction, and Soxhlet extraction. The influence of the type and time of isolation technique on the extraction process was studied, and the quality of the obtained extracts was determined by spectrophotometric and chromatographic methods to find the optimal extraction conditions. Radical scavenging activity of the extracts was analyzed using DPPH assay, while total phenolic content (TPC) was analyzed by the method with the Folin–Ciocalteu reagent. Application of gas chromatography with mass selective detector (GC-MS) enabled the identification of some bioactive substances and a comparison of the composition of the particular extracts. The Baikal skullcap root extracts characterized by both the highest antioxidant activity and content of phenolic compounds were obtained in 2 h of reflux and Soxhlet extraction. The main biologically active compounds identified in extracts by the GC-MS method were wogonin and oroxylin A, known for their broad spectrum of biological effects, including antioxidant, anti-inflammatory, antiviral, anticancer, and others.

## 1. Introduction

Both scientists and increasingly aware consumers are looking for natural raw materials that would have a health-promoting effect on the human body and could be used to diversify the daily diet. Very often, researchers’ attention focuses on plants that have been used for centuries, e.g., in local folk medicine in various parts of the world. Such plants are characterized by a specific chemical composition, which determines interesting and unique health-promoting properties. The biologically active compounds contained in them are mostly not used in modern medicine, which mainly uses the biologically active compounds obtained by chemical synthesis. Very often, individual biologically active substances are not as active as extracts obtained from plants with medicinal and nutritional properties. This is due to the fact that the presence of various compounds in the extract contributes to their synergistic effect, which enhances the health-promoting effects.

An example of such a plant is the Baikal skullcap (synonyms: Chinese skullcap, Hooded skullcap) (*Scutellaria baicalensis* Georgi). It is a perennial herbaceous plant from the family *Lamiaceae* whose root (*Radix scutellariae*) has been used in Chinese medicine for over 2000 years. This plant comes from Korea, China, the Russian regions of the Far East, Mongolia, and Siberia, but it has also been introduced into experimental crops in Europe, including Poland and Ukraine [1,2,3]. In its natural environment, at altitudes from 60 m to 2000 m above sea level, it grows on dry, sunny, and grassy slopes. In traditional Chinese medicine, this raw material was used in colds, lung and liver diseases, in the treatment of diarrhea, dysentery, hypertension, hemorrhages, insomnia, inflammation, and respiratory infections [4]. Typically, the roots are harvested in spring or autumn, then dried and used in their natural form or processed into powders, tinctures, or pills [4]. Roots contain many compounds of nutritional and pharmacological importance, with a total of about 130 compounds having been isolated so far [5]. The dominant compounds in the root are flavonoids and glycosides [6]. So far, over 40 different polyphenols have been identified, including flavonoids and their flavonols, dihydroflavones and their dihydroflavonols, chalcones, and bioflavonoids. Among them, the most characteristic of this plant’s raw material are baicalin, baicalein, wogonoside, and wogonin [6,7,8,9,10,11,12,13,14,15]. Biologically active compounds are found in the roots, with antioxidant and anti-inflammatory effects, among others, contributing to multidirectional health-promoting effects, as demonstrated by studies conducted on animal models (rats, mice), cell lines, and clinical trials. Baikal skullcap root extracts are characterized by, among others, the following effects: (1) anti-inflammatory [10,16,17,18,19,20], including within the pancreas [21], uterine mucosa [22], stomach [23], and lungs [24]; (2) antioxidant [2,7,25]; (3) hepatoprotective [26,27]; (4) protective for bones and joints [28,29,30] and the circulatory system [31]; (5) autoimmune [32]; (6) antiviral [33]; (7) antibacterial [34], including against bacteria causing tooth decay [35] and a resistance to antibiotics [36]; and (8) anti-cancer [37], e.g., in the case of nasopharyngeal tumors [38], cervical cancer [39], the liver [40], the large intestine [15], and ovaries [41].

Moreover, Yao et al. indicated the potential use of Baikal skullcap root in the prevention and treatment of Covid-19 [42]. Research by Ma et al. indicates that *Scutellaria baicalensis* may also alleviate depressive behaviors [43]. The potential of this raw material in the treatment of neurological diseases and improvement of cognitive functions [44,45,46], including Parkinson’s [47] and Alzheimer’s disease [48], has also been demonstrated.

The extraction process of bioactive compounds from plants depends on many factors, including the type of solvent used, the ratio of solvent to plant material, the applied extraction technique conditions, and all pre- and post-treatment operations. In the literature, there are some reports related to the extraction of Baikal skullcap root, conducted in various conditions. Ni et al. [49] described the extraction process of baicalin from *S. baicalensis* using water, optimized by orthogonal test and controlled by an HPLC analysis of the baicalin yield. An interesting, non-conventional approach has been described by Choi et al. [50], who applied alkaline reduced water as a solvent for Baikal skullcap extraction at 100 °C for 24 h, which resulted in a slight improvement of extraction efficiency and higher DPPH radical scavenging activity than using distilled water or 70% ethanol in similar conditions. Generally, among organic solvents, methanol and ethanol in various concentrations were described in the literature as the most effective solvents in the isolation of different flavonoids and other bioactive compounds from *S. baicalensis* [51,52,53]. As an alternative to the conventional organic solvents, natural deep eutectic solvents (NADES) were also proposed, and their effectiveness in the extraction of various flavonoids from this plant was compared to 70% ethanol and 80% methanol aqueous solutions [54]. An application of a supercritical fluid extraction (SFE) for the extraction of the flavonoids from the root of *S. baicalensis* was described as being preferable in comparison to the conventional extraction using ultrasounds [55]. However, the availability of this method is limited and the costs are high, as well as microwave-assisted extraction (MAE) [52].

The aim of this study was to investigate the influence of the isolation technique conditions on the antioxidant activity and content of bioactive compounds in *Scutellaria baicalensis* root ethanolic extracts and to indicate the best conditions for extraction and obtaining dry extracts of this plant. It was possible to conduct the comprehensive research by applying a uniform methodology for different extraction techniques in variable time: maceration, maceration with shaking, ultrasound-assisted extraction, reflux extraction, and Soxhlet extraction, which has not been described in the literature so far.

## 2. Results and Discussion

### 2.1. Antioxidant Potential of Baikal Skullcap Root Extracts

Five popular extraction techniques were used in studies, starting from the simple maceration (M) and maceration with shaking (MSH) to the more advanced and effective processes, including ultrasound-assisted extraction (UAE), reflux extraction (RE), and Soxhlet extraction (SE). All experiments were performed using 96% ethanol as a solvent in variable extraction time (0.5, 1, and 2 h). The results of antioxidant activity were measured spectrophotometrically using the DPPH method and expressed as radical scavenging activity (RSA) for extracts obtained in various conditions. They are shown in Table 1.

As can be seen in Table 1, the radical scavenging activity of Baikal skullcap root extracts obtained by the maceration process was relatively low and varied from 15.46% to 24.04%. Application of shaking resulted in an approximate doubling, while using ultrasounds almost quadrupled these values. However, the highest antioxidant activity was observed in the extracts obtained using reflux- and Soxhlet-extraction techniques, where, even for 0.5 h processes, the obtained RSA values exceeded 90%.

**Figure 1:**
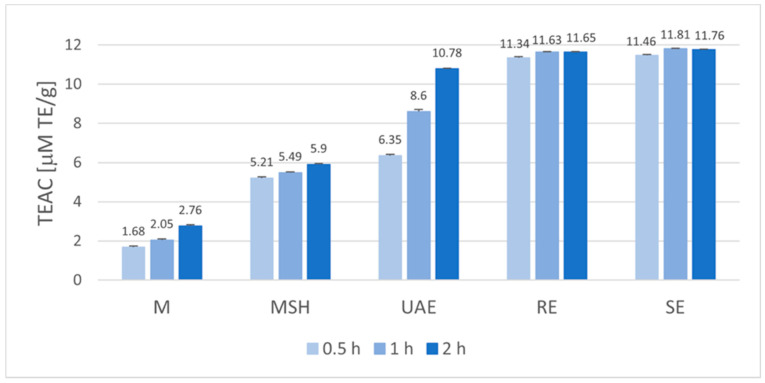
Influence of extraction technique and time on Trolox Equivalents Antioxidant Capacity (TEAC) of Baikal skullcap root ethanolic extracts (M—maceration; MSH—maceration with shaking; UAE—ultrasound-assisted extraction; RE—reflux extraction, SE—Soxhlet extraction).

**Figure 2:**
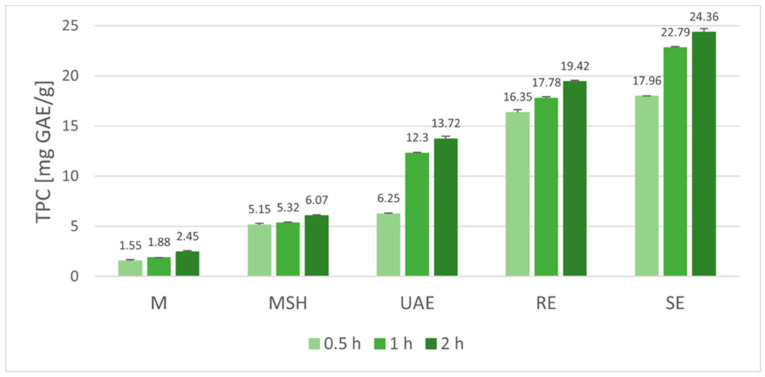
Influence of extraction technique and time on total phenolic content (TPC) of Baikal skullcap root ethanolic extracts (M—maceration; MSH—maceration with shaking; UAE—ultrasound-assisted extraction; RE—reflux extraction, SE—Soxhlet extraction).

In order to illustrate the tendencies observed and facilitate the proper interpretation of the results, the graphical visualization of the extraction conditions’ effect on the antioxidant activity and the total phenolic content were conducted. The antioxidant potential, expressed as the Trolox Equivalents Antioxidant Capacity (TEAC) for extracts obtained in various conditions, is presented in Figure 1, while the total phenolic content (TPC) was expressed in Gallic Acid Equivalents in Figure 2. Both parameters were calculated in relation to 1 g of plant material subjected to extraction in various conditions.

Analyzing the antioxidant activity of the obtained extracts (Figure 1), it was found that the TEAC values varied from 1.68 µM TE/g for 0.5-h maceration (M 0.5 h) to the similar highest values above 11 µM TE/g for all reflux- (RE) and Soxhlet- (SE) extraction processes. Generally, prolongation of the extraction time from 0.5 h to 1 h resulted in an increase of antioxidant potential in the case of all extraction techniques, but for maceration (M), maceration with shaking (MSH), reflux (RE), and Soxhlet (SE) extraction, this effect was not very strong. For the 2 h processes, observable enhancement was found in the case of maceration (2.76 µM TE/g) and maceration with shaking (5.90 µM TE/g), but especially for ultrasound-assisted extraction (10.78 µM TE/g). The influence of extraction time was the most noticeable for the UAE technique, which can be caused by the observable rise of the temperature from room temperature to 52 °C after 2 h. In the case of reflux (RE) and Soxhlet (SE) techniques, extending the extraction time from 1 h to 2 h did not result in a further increase in antioxidant activity.

Analyzing the total phenolic content in the extracts (Figure 2), some similarities can be seen between the observed trends in comparison to antioxidant activity. TPC values obtained for ethanolic extracts varied from 1.55 mg GAE/g (0.5 h M) to 24.36 mg GAE/g (RE 2 h). Regardless of the technique used, the phenolic content increased with prolongation of the extraction time from 0.5 to 2 h. The effectiveness of maceration was low, resulting after 2 h in TPC = 2.45 mg GAE/g for simple maceration (M 2 h) and 6.07 mg GAE/g when shaking was applied (MSH 2 h). Extracts obtained in a 0.5 h ultrasonic-assisted extraction (UAE 0.5 h) are also characterized by a relatively low content of phenolic compounds (6.25 mg GAE/g), but extending the time to 1 and 2 h allowed for the obtainment of a significantly higher effectiveness of phenolic compound extraction (12.30 and 13.72 mg GAE/g, respectively). Reflux extraction was an even more effective technique, resulting in TPC values ranging from 16.35 mg GAE/g (RE 0.5 h) to 19.42 mg GAE/g (RE 2 h). Nevertheless, the best technique for the isolation of phenolic compounds was Soxhlet extraction (SE), allowing for the obtainment of the TPC values from 17.96 mg GAE/g (0.5 h SE) to 24.36 mg GAE/g (2 h SE).

On the basis of both the antioxidant activity and total phenolic content measurements, it can be indicated that the most effective techniques were Soxhlet (SE 2 h) and reflux extraction (RE 2 h) conducted in the longest extraction time. The antioxidant potentials of extracts obtained in these conditions were similar (11.76 and 11.65 and µM TE/g, respectively). The application of 2 h of Soxhlet extraction allowed for the isolation of the highest amount of phenolic compounds (24.36 mg GAE/g), but the result obtained by using 2 h of reflux extraction was not much lower (19.42 mg GAE/g). Although there are some articles available in the literature related to Baikal skullcap extraction [49,50,51,52,53,54], no analogous studies were found, comparing the effectiveness of the various extraction techniques and conditions using 95% ethanol, which were the subject of this work. For this reason, as well as because other authors studied various parts of the plant, including roots and leaves [52] or hairy root culture [8], using different research methodologies and different units, it is difficult to compare the presented results with the others that can be found in the scientific publications.

### 2.2. Statistical Analysis of Antioxidant Potential and Total Phenolic Content

Descriptive statistics of the antioxidant potential of all analyzed Baikal skullcap root extracts, measured by the DPPH method (as RSA and TEAC) and total phenolic content (TPC), are summarized in Table 2.

Statistical analysis of the data showed that, for the particular time variants (0.5 h, 1 h, or 2 h), the differences in the antioxidant potential expressed as RSA (Table 1) and TEAC (Figure 1) between the various extraction techniques used were significant (*p* ≤ 0.05). The only exception was a statistically insignificant difference between the antioxidant properties of the extracts obtained in 0.5 h processes using the RE and SE techniques (*p* = 0.202). If we consider the individual extraction techniques, the antioxidant potentials of extracts obtained after 0.5 h, 1 h, and 2 h differed significantly in the case of M, MSH, and UAE techniques (*p* < 0.05). No significant differences were found in the antioxidant potential of extracts obtained after 1 h and 2 h using the RE and SE methods, (*p* = 0.874 and *p* = 0.325, respectively).

In the case of the total phenolic contents (Figure 2), statistical analysis of the data showed that, for all particular time variants (0.5 h, 1 h, and 2 h), the differences between TPC for the extracts obtained by the various extraction techniques were significant (*p* < 0.05). Importantly, considering the individual techniques of extraction, data for the extracts obtained after 0.5 h, 1 h, and 2 h differed significantly (*p* < 0.05) for each applied method (Figure 2).

The correlations between the parameters studied, including antioxidant potential (TEAC), total phenolic content (TPC), and extraction time for each extraction technique, were also statistically analyzed (Table 3). In most cases (M, MSH, UAE, and RE), a highly significant correlation was found between extraction time and antioxidant potential (correlation coefficient (r) between 0.76 and 0.99). This indicates that the prolongation of the extraction process led to higher values of the antioxidant potential. The only exception was the Soxhlet-extraction (SE) technique, where no significant effect of extraction time on the antioxidant potential was shown. Importantly, regardless of the extraction technique used, positive significant correlations were found between extraction time and total phenolic content (correlation coefficient (r) between 0.85 and 0.97). Furthermore, the statistical correlation between antioxidant potential and total phenolic content (TEAC vs. TPC) was also investigated. For each extraction technique used, positive significant correlations were found between these parameters (correlation coefficient (r) between 0.85 and 0.98). It means that the antioxidant potential of the extracts, measured as the DPPH scavenging activity, may result from the content of various hydroxyflavones (including identified wogonin and oroxylin A) and other compounds belonging to the group of phenolic compounds. High correlation between DPPH scavenging activity and TPC was described in the literature by other authors who studied various plants [56,57].

The performed statistical analysis confirmed that due to significant differences between the particular extraction conditions, the reflux and Soxhlet extraction performed in the longest extraction time (2 h) allowed for the obtainment of the extracts that were characterized by both the highest antioxidant potential and total phenolic content. Therefore, these conditions can be indicated as the best among all that were applied in the studies.

### 2.3. Dry Extracts of Baikal Skullcap Root

For the best extraction conditions selected on the basis of analyses, with the results described above, the research was extended by obtaining the dry extracts from *S. baicalensis* root, determining their yields and antioxidant properties. After removal of the solvent, gentle drying at 40 °C, and stabilization in the desiccator, the yields of dry extracts were calculated considering the obtained masses. The next step was determining the half-maximal inhibitory concentration, the IC_50_ parameter, which required preparing the series of solutions of the known concentrations and analyzing their RSA [%] values. The values of IC_50_, which inversely correlated with the radical scavenging activity, were found from the RSA = f(C) equation in the linear concentration range (0.1–0.5 mg/mL). The yields and antioxidant properties of dry extracts obtained by 2 h of reflux extraction and Soxhlet extraction are compared in Table 4.

Analyzing the results obtained, it can be noticed that 2 h of Soxhlet extraction of Baikal skullcap root using ethanol allowed for the obtainment of a higher yield of dry extract (21.7%) than reflux extraction (17.0%). The extract obtained in the Soxhlet apparatus was characterized by a slightly higher antioxidant potential (lower value of IC_50_) than obtained by boiling under reflux, but, in fact, their values of IC_50_ are very similar (0.37 and 0.39 mg/mL, respectively). Comparing the obtained yields of extraction to the results of other authors who obtained 20.3% yield during extraction with 70% ethanol at 80 °C for 24 h [50], it can be noticed that an application of 95% ethanol in our research enabled the achievement of similar results in a much shorter time (2 h). Generally, it can be stated that the extracts obtained using these two different techniques were obtained with good yields, and that they were characterized by similar antioxidant potential. Nevertheless, considering practical aspects related to the possible scale-up of the process of obtaining of dry extracts, the use of extraction at the boiling temperature under reflux may be more convenient than the use of the Soxhlet apparatus.

### 2.4. GC-MS Analysis

**Figure 3:**
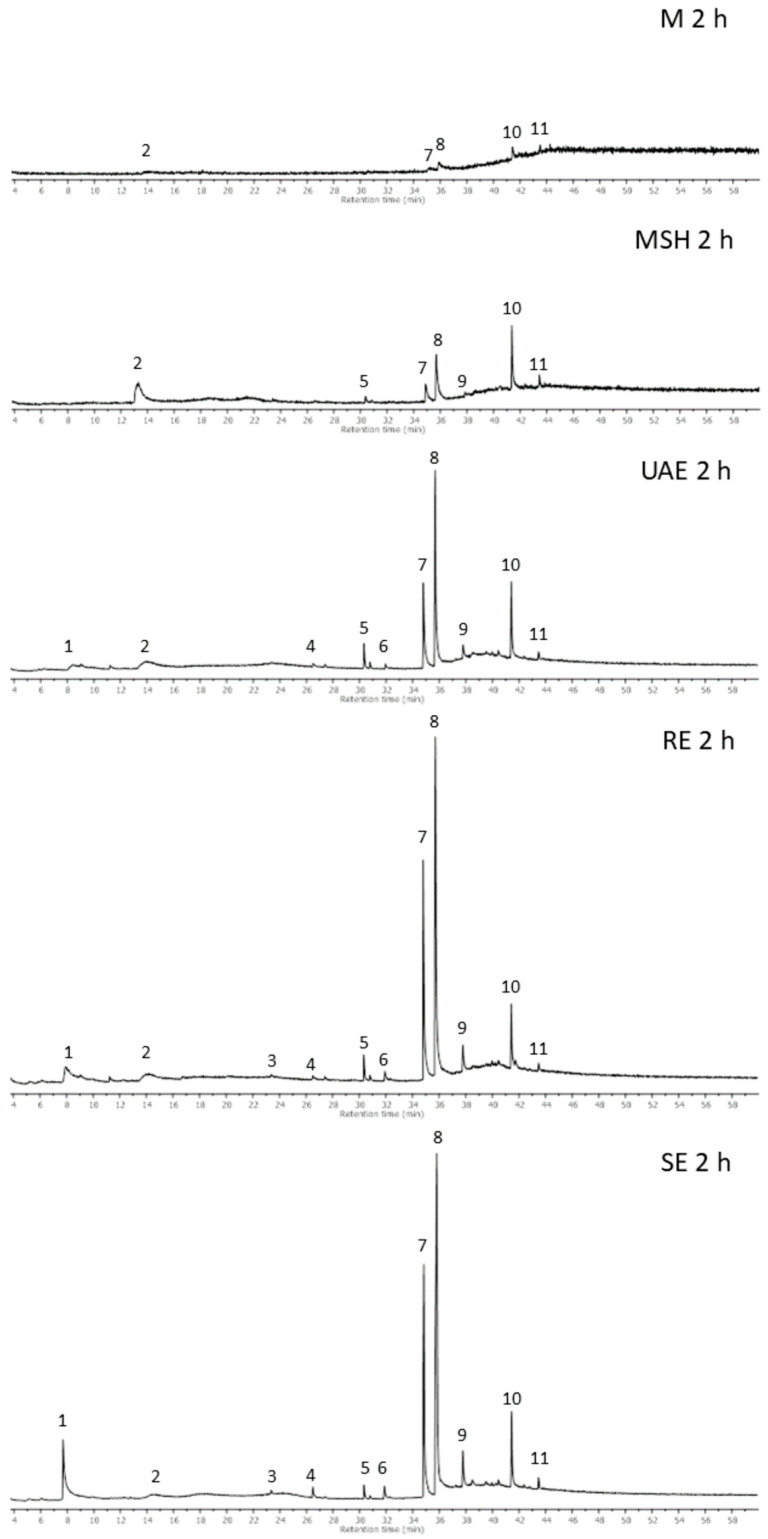
Total Ion Chromatograms of ethanolic extracts of Baikal skullcap (*S. baicalensis*) root obtained by various extraction techniques (peaks numbered according to Table 5).

**Figure 4:**
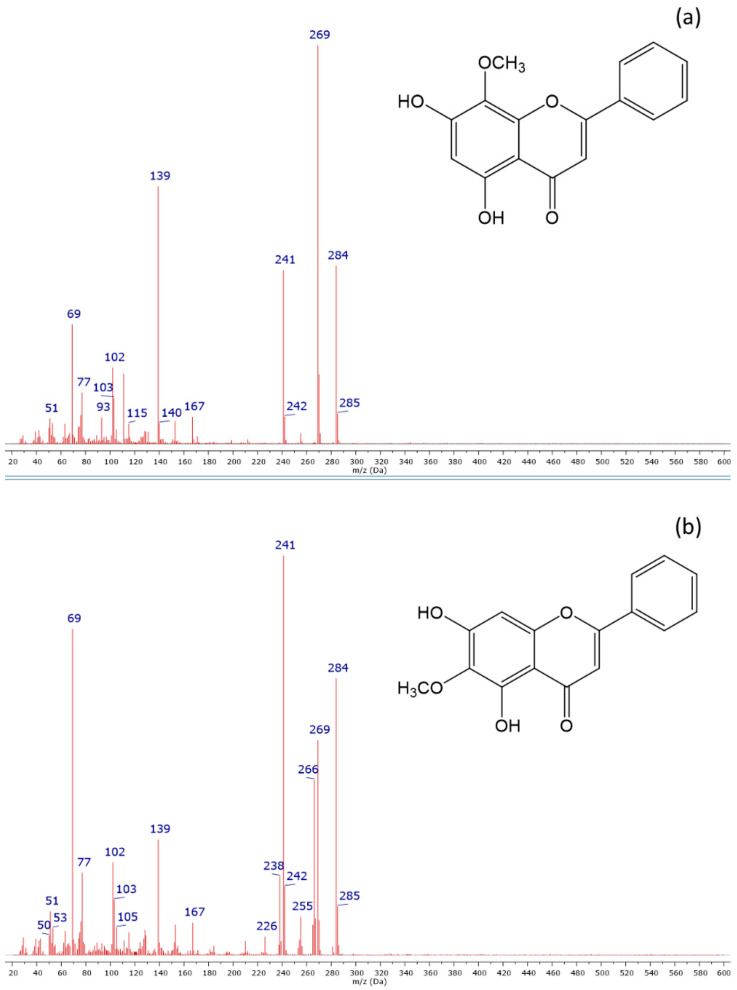
Mass spectra of wogonin (**a**) and oroxylin A (**b**) identified in ethanolic extracts of Baikal skullcap (*S. baicalensis*) root.

The composition of Baikal skullcap root ethanolic extracts was analyzed by gas chromatography with the mass selective detector (GC-MS) method. The Total Ion Chromatograms (TICs) of the extracts obtained by 2 h of extraction using all applied techniques are shown in Figure 3, while the obtained chromatographic data are summarized in Table 5. GC-MS analysis allowed for the identification of nine compounds, which were present in the extracts and for the comparison of their contents, although some compounds remained unidentified (Peaks No. 2 and 6), and some others are not possible to analyze using this method, including the non-volatile and very polar compounds. The mass spectra of the main bioactive compounds characteristic of the Baikal skullcap, which were identified in the extracts (wogonin and oroxylin A), are shown in Figure 4. The composition of dry extracts was also analyzed by the GC-MS method, and it was found to be in good agreement with the composition of the primary ethanolic extracts obtained in the same conditions.

The main components found in the extracts were wogonin and oroxylin A (Figure 4), belonging to the flavonoid compounds characteristic of the Baikal skullcap [12,13]. The identification of wogonin was performed by comparison of the mass spectrum with the data of wogonin from the NIST 04 library, while its isomer, oroxylin A, was identified based on the comparison with the mass spectrum presented in the literature [58]. In addition to these main compounds, two other compounds, also belonging to the flavonoids, were identified based on the mass spectra analysis: dihydroxydimethoxyflavone and dihydroxytetramethoxyflavone. Their mass spectra show similarity to the mass spectra of wogonin and oroxylin A (M = 284), and the differences in the molecular masses probably correspond to additional 1 and 3 methoxy groups (CH_3_O-) in the structure of dihydroxydimethoxyflavone (M = 314) and dihydroxytetramethoxyflavone (M = 374). The presence of these compounds in the Baikal skullcap was described in the literature [59]. Unfortunately, the exact determination of the positions of the particular substituents was not possible and requires further in-depth analysis.

The compounds from the flavonoid group belong to popular antioxidants, and their presence and amounts are often correlated with the antioxidant properties of the plant extracts. In our studies, it was also observable that the increased amounts of flavonoids were correlated with the rise of antioxidant potential, and that their highest contents were found in the optimized extraction conditions: RE 2 h and SE 2 h. Comparing the composition of these extracts (Figure 3, Table 5), a big similarity can be seen. In addition to the described flavonoids, these extracts contained small amounts of biologically active compounds, such as β-sitosterol and (Z)-9-octadecenamide, as well as trace amounts of linoleic and palmitic acid.

As a result, extracts rich in compounds with high and interesting biological activity were obtained, mainly wogonin and oroxylin A, characterized by a broad spectrum of action. Wogonin is known for its anti-inflammatory, antiviral, anticancer, antioxidant, and hepatoprotective effects [6,7,8,9,10,11,12,13,14,15], and oroxylin A is attributed with the anti-inflammatory, antioxidant, and strongly neuroprotective properties [60]. β-Sitosterol lowers cholesterol levels and has high antioxidant and anti-inflammatory activity [61]. Linoleic acid is known for its hypolipidemic antioxidant and antiglycemic effects [62], while (Z)-9-octadecenamide has anxiolytic and hypnotic properties, and, therefore, it can be used in preparations that facilitate falling asleep [63].

Unexpectedly, in the most severe extraction conditions, the presence of 5-hydroxymethylfurfural (HMF) was also found, with the largest amount in the extract being obtained during 2 h of extraction in the Soxhlet apparatus. This compound is probably not a metabolite of the tested plant because it is known from the literature that it is formed during the processing and storage of various foods and plants rich in reducing sugars and aminoacids as the product of the Maillard reaction [64]. There are contradictory reports in the literature regarding the activity and toxicity of HMF. According to some reports, it may be responsible for cytotoxic and carcinogenic effects [65], while other researchers believe that it does not pose a serious health risk and may even possess valuable properties, such as antioxidant, antiproliferative, anti-inflammatory, or anti-allergic [66,67]. In our studies, its formation may be due to the effect of the high temperature associated with the use of an electric bath during extraction in the Soxhlet apparatus, in contrast to extraction under reflux, where a water bath was used and a significantly smaller amount of this compound was found. This leads to an important practical premise to avoid any possible overheating during the extraction process, which can lead to the formation of this by-product.

Considering all of the results obtained, for the larger-scale production of dry extracts from Baikal skullcap, 2 h of reflux extraction with ethanol, using a water bath or other effectively controlled sources of heat, can be recommended. The extracts obtained in these conditions were characterized by similar high antioxidant potential and chemical composition in comparison to 2 h of Soxhlet extraction. The yield of the dry extract was a little lower than using the Soxhlet apparatus, but the formation of by-product 5-hydroxymethylfurfural was significantly reduced.

## 3. Materials and Methods

### 3.1. Plant Material

Commercially available finely ground root of Baikal skullcap (*Radix Scutellariae baicalensis*) was used for the studies. The plant material originated from Poland (producer: FARMVIT, Szczecin, Poland). A form of intake suggested by producer is infusion prepared by boiling 2–3 g in 200 mL of water for 5 min, leaving for 30 min, and straining the obtained herbal tea. For better extraction of the bioactive compounds, previous generous sprinkling of plant material using 96% ethanol is recommended by the producer.

### 3.2. Extraction Techniques and Sample Preparation

Baikal skullcap root was extracted using 96% ethanol as a solvent (Stanlab, Poznań, Poland). Ethanol, being a safer alternative than toxic methanol, was selected as a good solvent for the extraction of bioactive compounds from Baikal skullcap, which are mainly polar compounds [51,52,53,54,55]. Using ethanol in a concentration of 96% as the extractant can also play an additional role in plant material sterilization. Five various extraction techniques were applied in studies: maceration (M), maceration with shaking (MSH), ultrasound-assisted extraction (UAE), reflux extraction (RE), and Soxhlet extraction (SE). For each technique used, the influence of the extraction time was also studied by performing processes by 0.5 h, 1 h, and 2 h. In the majority of the experiments, 5.00 g of Baikal skullcap root and 50 mL of solvent (96% ethanol) were used. The only exception was Soxhlet Extraction (SE), where the same proportions of material to solvent were applied, but their amounts were doubled (10.00 g and 100 mL, respectively) in order to adjust them to the size of the Soxhlet extractor. Maceration (M) and maceration with shaking (MSH) processes were conducted at room temperature (23 °C) in the 50 mL round bottom flasks equipped with ground glass stoppers. Reflux extraction (RE) and ultrasound-assisted extraction (UAE) were performed in the 50 mL round bottom flasks equipped with the reflux condensers, using a water bath (temperature setting: 95 °C) and a Sonis 4 (Iskra PIO, Šentjerna, Slovenia) ultrasonic bath (power: 75 W, frequency: 40 kHz). During UAE extraction, the temperature in ultrasonic bath increased from room temperature to 35 °C (0.5 h), 44 °C (1 h), and 52 °C (2 h), respectively. The electric bath (temperature setting: 120 °C) was used for heating during extraction in Soxhlet apparatus. After completing the extraction process, the samples were cooled using running water to room temperature and left for 5 min for sedimentation of a plant material followed by decantation. The decanted extracts were subjected to centrifugation for 10 min at 2320 RCF using MPW-223e Centrifuge (MPW Med. Instruments, Warszawa, Poland). Finally, 0.5 mL of the obtained clear extracts were filled up with ethanol to 10 mL in volumetric flask to prepare the samples of extracts for the analyses.

In the case of the extracts that were characterized by the best properties, dry extracts were additionally obtained and subjected to further research. For this purpose, the extracts were placed in Petri dishes and left under the fume hood for solvent evaporation at room temperature, protecting them from the light. Next, they were dried for 16 h at 40 °C using a laboratory dryer and then stabilized in the desiccator to a constant mass. The yields of the dry extracts were calculated from the relation of their masses to the masses of raw material used in the experiments.

### 3.3. Antioxidant Activity

The antioxidant properties of the extracts obtained from Baikal skullcap in various conditions were studied spectrophotometrically by means of DPPH radical scavenging assay [68]. The analyses were performed using a 1600PC UV–VIS spectrophotometer (VWR International, Leuven, Belgium) in 1 cm glass cuvettes. A radical scavenging activity of extracts was determined using the DPPH method with a 2,2-diphenyl-1-picrylhydrazyl radical (DPPH). Before the analysis, 0.002 mM/mL of DPPH stock solution in methanol was prepared, and then 3 mL of this solution were diluted with methanol to 50 mL in a volumetric flask in order to obtain a DPPH working solution. All DPPH solutions were freshly prepared and protected from light by using aluminum foil. Next, 3 mL of the DPPH working solution were added to 0.5 mL of the prepared sample of extract, mixed, and left for incubation in darkness for exactly 30 min. All analyses were conducted in three repetitions, and the absorbance was measured at 517 nm. A reference sample containing 0.5 mL of a solvent (96% ethanol) was prepared and analyzed analogously. The radical scavenging activity (RSA) of Baikal skullcap extracts was calculated from the absorbance of the sample (A_30_) and the absorbance of reference sample A_0_, as follows:

RSA [%] = 100 (A_0_ − A_30_)/A_0_, (1)

The DPPH scavenging activity of the samples extracted in various conditions was also expressed as Trolox Equivalents Antioxidant Capacity (TEAC). For this purpose, the various concentrations of Trolox (Acros Organics, Geel, Belgium) standard solutions were prepared and analyzed analogously. On the basis of the obtained results, the calibration curve of RSA [%] versus C_Trolox_ [µM/L] was prepared. TEAC values for the studied extracts were calculated from the obtained linear regression equation in the concentration range of linearity (10–300 µM/L) and, finally, expressed in relation to 1 g of extracted plant material [µM TE/g].

Moreover, for the dry extracts obtained in the optimized conditions, the IC_50_ parameter was determined, referred to as the half-maximal inhibitory concentration, which results in 50% inhibition of the free radical activity. For the antioxidant activity tests of dry extracts, stock solutions in ethanol (C = 10 mg/mL) were first prepared and diluted with ethanol in volumetric flasks to obtain the series of working solutions in the concentration range of 0.1–0.9 mg/mL. On the basis of the analysis of their RSA [%] values, the linear range was estimated (from 0.1 to 0.5 mg/mL), which allowed for the determination of the IC_50_ parameter from the equation of the obtained trend line.

### 3.4. Total Phenolic Content (TPC)

Folin–Ciocalteu (F–C) method [69] was applied for spectrophotometric determination of the total amount of phenolic compounds extractable from the plant material by using ethanol in various conditions. For this purpose, 0.5 mL of the prepared samples of extracts, 0.5 mL of a Folin–Ciocalteu reagent (Chempur, Piekary Śląskie, Poland), and 1.5 mL of freshly prepared sodium carbonate (Na_2_CO_3_) solution in water (200 mg/mL) were placed in a 25 mL volumetric flask and filled up with a demineralized water. The solutions were mixed and left for 30 min, while the development of the blue color could be observed. Next, the absorbance at 760 nm was measured for the extract samples and the reference samples, which contained only solvents and reagents (all analyses in three repetitions). The calibration curve was prepared using gallic acid (GA) as a standard (Sigma–Aldrich, St. Louis, MO, USA) on the basis of analyses of standard solutions in a concentration range of 20–400 mg/L performed using the same procedure. The total phenolic content (TPC) was expressed as mg of Gallic Acid Equivalent extracted from 1 g of the plant material [mg GAE/g].

### 3.5. GC-MS Analysis

In order to identify the biologically active substances in the ethanolic extracts of Baikal skullcap, the gas chromatography with mass selective detector (GC-MS) method was applied. The analyses were performed using a 6890N gas chromatograph with a 5973 Network Mass Selective Detector (Agilent Technologies, Palo Alto, CA, USA) equipped with an HP-5MS capillary column (5%-phenyl 95%-methylpolysiloxane, 30 m × 0.25 mm × 0.25 μm). The column temperature was programmed and increased from 80 °C at 5 °C/min to 300 °C (kept by 16 min). The MSD temperatures were as follows: quadrupole 150 °C and ion source 230 °C. The carrier gas was helium (1.2 mL/min). The samples of extracts (3.0 μL) were injected into a column in a split mode (10:1) using a 7683 Series Injector Autosampler. Electron impact ionization (70 eV) mass spectra were recorded via SCAN mode in the range of 20–600 *m*/*z*. Identification of the particular compounds was conducted by the comparison of their mass spectra with the data of available standards, standards from the NIST 04 library, and for oroxylin A with the literature data [58]. The identification was confirmed by the comparison of the calculated linear retention indices (RI) with the values found in the literature [70] when the data were available. To determine the retention indices, the standard mixture of the C_7_-C_40_ n-alkanes (1000 μg/mL) in heksane (Supelco, Bellefonte, PA, USA) was analyzed under the same chromatographic conditions. In the case of dihydroxydimethoxyflavone and dihydroxytetramethoxyflavone, the identification was performed on the basis of the analysis of characteristic ions in the mass spectrum. The estimated contents of particular compounds were found based on their peak area percentage in the Total Ion Chromatogram (TIC) of the extracts obtained using the MestReNova 10.0.2 software.

### 3.6. Statistical Analysis

Statistical analysis was performed using StatSoft Statistica 13.0 (STATISTICA 13.0; StatSoft Inc., Palo Alto, CA, USA) and Microsoft Excel 2021. In all the experiments, three samples were analyzed, and all the assays were conducted at least in triplicate. The results are expressed as the mean values and standard deviation (mean ± SD). For antioxidant potential and total phenolic content, one-way analysis of variance (ANOVA) and Tukey post-hoc test were used. Correlation analysis was performed by *Pearson* coefficient. Differences were considered significant at *p* ≤ 0.05.

## 4. Conclusions

Our studies of Baikal skullcap root ethanolic extracts obtained in various conditions enabled the determination of the impact of extraction conditions on their DPPH radical scavenging activity and total phenolic content. The effectiveness of the simple maceration process was low, and the enhancement possibilities of this process by shaking and application of the ultrasounds were not as effective as the application of Soxhlet and reflux extraction. The extracts obtained using these two techniques were characterized by high-antioxidant properties and content of valuable compounds, mainly wogonin and oroxylin A, possessing a broad spectrum of bioactive properties. The results of these studies can be applied in the development and optimization of extraction processes of active ingredients from Baikal skullcap roots in the production of dietary supplements characterized by the high-antioxidant potential and content of health-promoting compounds. On the basis of the results obtained, 2 h of reflux extraction using ethanol can be recommended as the convenient and effective technique for the larger-scale production of dry extracts from this precious plant.

## Figures and Tables

**Table 1:** Radical scavenging activity (RSA) of Baikal skullcap (*S. baicalensis*) root extracts obtained using ethanol as a solvent in various conditions.

Extraction Technique	Extraction Time [h]	RSA [%] (Mean ± SD *)
Maceration (M)	0.5	15.46 ± 0.50
	1	18.40 ± 0.43
	2	24.04 ± 0.52
Maceration with shaking (MSH)	0.5	43.42 ± 0.47
	1	45.65 ± 0.27
	2	48.85 ± 0.46
Ultrasound-assisted extraction (UAE)	0.5	52.41 ± 0.53
	1	70.19 ± 0.80
	2	87.46 ± 0.22
Reflux extraction (RE)	0.5	91.88 ± 0.50
	1	94.16 ± 0.25
	2	94.30 ± 0.13
Soxhlet extraction (SE)	0.5	92.82 ± 0.43
	1	95.59 ± 0.18
	2	95.22 ± 0.17

* SD–standard deviation (n = 3).

**Table 2:** Descriptive statistics of the tested variables of antioxidant potential and total phenolic content for Baikal skullcap root extracts.

Variables (n = 45)	Mean	Median	Min.	Max.	Lower Quartile	Upper Quartile	Standard Deviation
RSA [%]	64.656	70.500	14.936	95.763	43.921	93.978	29.942
TEAC [µM TE/g]	7.898	8.637	1.610	11.832	5.275	11.606	3.786
TPC [mg GAE/g]	11.535	12.260	1.414	24.630	5.191	17.937	7.702

**Table 3:** Correlation coefficients (r) between tested parameters.

Extraction Technique	TEAC vs. Time	TPC vs. Time	TPC vs. TEAC
Maceration (M)	0.9937 *	0. 9715 *	0.9869 *
Maceration with shaking (MSH)	0.9849 *	0.9579 *	0.9641 *
Ultrasound-assisted extraction (UAE)	0.9798 *	0.8597 *	0.9427 *
Reflux extraction (RE)	0.7651 *	0.9663 *	0.8515 *
Soxhlet extraction (SE)	0.6570	0.8871 *	0.9166 *

* Significant differences at *p* ≤ 0.05.

**Table 4:** Characteristic of dry extracts obtained from Baikal skullcap (*S. baicalensis*) root using ethanol as a solvent in the optimized conditions.

Extraction Conditions	Yield [%]	C [mg/mL]	RSA [%]	RSA(y) vs. C(x) Equation	R^2^	IC_50_ [mg/mL]
RE 2 h	17.0	0.1 0.2 0.3 0.4 0.5	12.21 25.35 38.58 52.10 63.28	y = 128.89x − 0.363	0.9990	0.39
SE 2 h	21.7	0.1 0.2 0.3 0.4 0.5	14.19 25.35 38.58 52.10 63.28	y = 127.95x + 2.393	0.9982	0.37

**Table 5:** Compounds identified in ethanolic extracts of Baikal skullcap (*S. baicalensis*) root by GC-MS analysis.

No.	Compound	RT [min]	RI	Identification Methods	MS Signals *, *m/z*		Presence in Extract **				
					[M^●^]^+^	Characteristic Fragment Ions	M	MSH	UAE	RE	SE
1	5-Hydroxy-methylfurfural	7.72	1251	MS-NIST	126	97, 41, 126, 69, 29	nd	nd	tr	+	++
2	unidentified	14.05	1524	-	164(?)	31,29,57, 43, 73	tr	+	+	+	tr
3	Palmitic acid	23.36	1964	RI, MS-NIST	256	43,73, 60, 41, 57	nd	nd	nd	tr	tr
4	Linoleic acid	26.49	2133	RI, MS-NIST	280	67,81, 82, 95, 55	nd	nd	nd	tr	+
5	(Z)-9-Octadecen-amide	30.35	2360	MS-NIST	281	59,72,55, 41, 43	nd	tr	+	+	+
6	unidentified	31.90	2456	-	286(?)	167, 69, 182, 78, 103	nd	nd	tr	tr	+
7	Oroxylin A	34.83	2649	MS-LIT	284	241, 69, 269, 266, 139	tr	+	++	+++	+++
8	Wogonin	35.80	2716	MS-NIST	284	269, 139, 241, 69, 167	tr	++	+++	+++	+++
9	Dihydroxydimethoxyflavone	37.78	2858	MS-analysis	314	299, 271, 169, 69, 102	nd	tr	+	+	++
10	Dihydroxytetramethoxyflavone	41.44	3139	MS-analysis	374	359,211,183,69, 127	tr	++	++	++	++
11	β-Sitosterol	43.47	3306	RI, MS-NIST	414	43,57,55,41,145	tr	tr	+	+	+

* Base peak ion underlined; (?)—supposed molecular ion; ** presence in extract, based on peak area in total ion chromatogram of extracts obtained in 2 h processes of maceration (M), maceration with shaking (MSH), ultrasound-assisted extraction (UAE), reflux extraction (RE) and Soxhlet extraction (SE): nd—not detected, tr—trace signal, +—minor component, ++—intermediate component, +++—major component; RT—retention time; RI—linear retention index determined experimentally on HP5-MS column; MS-NIST—match of mass spectrum (>95%) with the NIST 04 Library; MS-LIT—match of mass spectrum with presented in the literature [58]; MS-analysis—self-reliant analysis and interpretation of mass spectrum.

## Data Availability

The raw data supporting the conclusions of this article will be made available by the authors upon request.
